# Anti-Inflammatory Effects of the LK5 Herbal Complex on LPS- and IL-4/IL-13-Stimulated HaCaT Cells and a DNCB-Induced Animal Model of Atopic Dermatitis in BALB/c Mice

**DOI:** 10.3390/pharmaceutics16010040

**Published:** 2023-12-27

**Authors:** Hyun-Jeong Kim, So-Yeon Kim, Ho Jung Bae, Yu-Yeong Choi, Ju-Yeon An, Ye Eun Cho, So-Young Cho, Su-Jung Lee, Sanghyun Lee, MinSub Sin, Young Min Yun, Jong Ryul Lee, Se Jin Park

**Affiliations:** 1 Department of Food Biotechnology and Environmental Science, Kangwon National University, Chuncheon 24341, Republic of Korea; hjkim0702@kangwon.ac.kr (H.-J.K.); tyler441@kangwon.ac.kr (Y.-Y.C.); anjuyeon0907@kangwon.ac.kr (J.-Y.A.); 202215525@kangwon.ac.kr (Y.E.C.); whthdud67@kangwon.ac.kr (S.-Y.C.); 202316424@kangwon.ac.kr (S.-J.L.); 2 Agriculture and Life Science Research Institute, Kangwon National University, Chuncheon 24341, Republic of Korea; baehj321@kangwon.ac.kr; 3 Department of Plant Science and Technology, Chung-Ang University, Anseong 17546, Republic of Korea; slee@cau.ac.kr; 4 LK Co., Ltd., Hwaseong 18469, Republic of Korea; useless79@lkskincare.co.kr (M.S.); secret31.yun@lkskincare.co.kr (Y.M.Y.); jrlee01@lkskincare.co.kr (J.R.L.); 5 School of Natural Resources and Environmental Sciences, Kangwon National University, Chuncheon 24341, Republic of Korea

**Keywords:** LK5 herbal complex, atopic dermatitis, signal transducers and activators of transcription, itching, anti-inflammation

## Abstract

Atopic dermatitis (AD) is a chronic inflammatory skin disease influenced by a complex interplay of genetic and environmental factors. The activation of the JAK-STAT pathway increases the expression of inflammatory cytokines such as IL-4 and IL-13, further deteriorating AD. Therefore, for the treatment of AD, the JAK-STAT pathway is emerging as a significant target, alongside inflammatory cytokines. This study investigates the potential therapeutic effects of a novel herbal complex, LK5, composed of *Scutellaria baicalensis*, *Liriope platyphylla*, *Sophora flavescens*, *Dictammus dasycarpus*, and *Phellodendron schneider*, known for their anti-inflammatory and immune-modulating properties. We examined the anti-inflammatory and anti-AD effects of the LK5 herbal complex in HaCaT cells stimulated by LPS and IL-4/IL-13, as well as in a mouse model of AD induced by DNCB. In HaCaT cells stimulated with LPS or IL-4/IL-13, the LK5 herbal complex demonstrated anti-inflammatory effects by inhibiting the expression of inflammatory cytokines including TNF-α, IL-6, and IL-1β, and downregulating the phosphorylation of STAT proteins. In a murine AD-like model induced by DNCB, administration of the LK5 herbal complex significantly ameliorated clinical symptoms, including dermatitis, ear thickness, and TEWL. Histological analysis revealed a reduction in epidermal thickness and mast cell infiltration. The LK5 herbal complex also inhibited pruritus induced by compound 48/80. Furthermore, the LK5 herbal complex treatment significantly decreased the levels of inflammatory cytokines such as TSLP, IL-6, and IgE in plasma and ear tissue of AD-induced mice. These findings suggest that the LK5 herbal complex may modulate the immune response and alleviate AD symptoms by inhibiting STAT pathways.

## 1. Introduction

Atopic dermatitis (AD), characterized by inflammatory skin diseases, is known to occur mainly in infants and toddlers, but recently it was also found to occur in adulthood [1,2,3]. The pathophysiology of AD is not entirely understood, but it is caused by a combination of environmental and genetic factors [4,5]. External antigens stimulate T cells to produce thymic stromal lymphopoietin (TSLP), which induces T cells to differentiate into T helper 2 (Th2) cells, and IL-4 and IL-13 are expressed by Th2 cells [6,7]. IL-4 and IL-13, which are expressed by Th2 cells, stimulate B cells to produce immunoglobulin E (IgE) antibodies, histamines, and inflammatory cytokines released from mast cells by IgE [8,9]. The Janus kinases (JAK)-signal transducer and activator of transcription (STAT) pathway activated by IL-4 and IL-13 plays a pivotal role in the pathogenesis of AD [10]. Although, the JAK-STAT pathway is mainly known to cause cancer, it disrupts skin barriers and eventually induces worsened AD by increasing IgE production as well as the expressions of inflammatory cytokines such as IL-25 and IL-33 [11,12]. Moreover, STAT inhibitors such as tofacitinib and anti-IL-4/IL-13 monoclonal antibodies including dupilumab, were recently approved for medication against AD [13,14]. In addition, AD is accompanied by severe itching and redness [15]. The skin of AD patients is infiltrated with abnormal inflammatory cytokines and increased IgE and immune cells, such as macrophages, eosinophils, and mast cells [6]. Consequently, it is well documented that patients with AD experience increased epidermal thickness in the outermost layer of the skin, along with persistent skin inflammation and dryness [15]. 

AD is difficult to cure because it quickly recurs and repeats depending on the environment and personal conditions [16]. The most commonly used treatments for AD are steroids and antihistamines, which prevent skin damage by decreasing inflammation and itching [17]. Since side effects may occur when used for a long time, the application of steroids and antihistamine is limited [18]. Therefore, the need for medication with fewer side effects has increased, and various studies are being conducted using natural products such as plants and foods [19].

*Scutellaria baicalensis* belongs to Lamiaceae, and is known to control the immune system and have antioxidant and antibacterial effects [20]. *Liriope platyphylla* has been found to have anti-inflammatory effects, and *Sophora flavescens*, belonging to the Fabaceae, is used for eczema. Substances such as flavonoids and alkaloids have been separated, and further research has been conducted [21,22]. *Dictammus dasycarpus*, which belongs to Rutaceae, is the skin of the white pine tree root; it has anti-allergy and antifungal effects, since it contains components such as dictamnine and lemonine [20,23]. The anti-inflammatory and antibacterial properties of *Phellodendron schneider* have also been reported previously [24]. In this study, we hypothesized that the LK5 herbal complex composed of *Scutellaria baicalensis*, *Liriope platyphylla*, *Sophora flavescens*, *Dictammus dasycarpus*, and *Phellodendron schneider* with anti-inflammatory and anti-allergic effects would exhibit anti-AD effects. Based on this hypothesis, the LK5 herbal complex was investigated for its anti-inflammatory effects in vitro, in LPS or IL-4/IL-13-stimulated HaCaT cells. We also determined whether the LK5 herbal complex exhibited inhibitory effects on AD development in a DNCB-induced AD-like animal model.

## 2. Materials and Methods 

### 2.1. Animals

Six-week-old female mice were purchased from Orient Co., Ltd. (Seongnam, Republic of Korea). During the experimental period, the environment was maintained at a temperature of 23 ± 2 °C, humidity of 50 ± 10%, and a 12-h light–dark cycle (06:00–18:00). The experimental animals were provided with free access to solid feed (2018S; Envigo, Madison, WI, USA) and water from a tap. This animal test was conducted with the approval of the Institutional Animal Care and Use Committee of Kangwon National University (KW-200903-2).

### 2.2. Materials

The LK5 herbal complex (Lot NO. S-LKG2-p-210521) was donated by LK Co., Ltd. (Hwaseong, Republic of Korea). The LK5 herbal complex was a mixture of five plants, (*Scutellaria baicalensis*, *Liriope platyphylla*, *Sophora flavescens*, *Dictammus dasycarpus*, and *Phellodendron schneider*), extracted with 30% ethanol at 96 °C and spray-dried. Acetone, isopropyl alcohol, and olive oil were purchased from Daejung (Seongnam, Republic of Korea). Griess reagent, lipopolysaccharides from *Escherichia coli* O26:B6 (LPS), 3-(4,5-dimethylthiazol-2-yl)-2,5-diphenyl tetrazolium bromide (MTT), DNCB, compound 48/80, dexamethasone, terfenadine, and 10% formalin solution were purchased from Sigma-Aldrich Co. (St. Louis, MO, USA). RNA isoPlus and Dulbecco’s modified Eagle’s medium (DMEM), Dulbecco’s phosphate-buffered saline (DPBS), and penicillin–streptomycin (P/S) were procured from Takara Bio Inc. (Kusatsu, Japan) and Welgene (Gyenogsan, Republic of Korea), respectively. Fetal bovine serum (FBS) was purchased from Atlas Biologicals (Fort Collins, CO, USA). TransScript^®^ All-in-one First-Strand cDNA Synthesis SuperMix for qPCR (One-Step cDNA Removal) was purchased from TransGen Biotech Co. (Beijing, China). PowerSYBR^®^ Green PCR Master Mix from Applied Biosystems was purchased from Thermo Fisher Scientific (Rockford, IL, USA). IL-1β, IL-6, TNF-α, iNOS, TSLP, IL-4, IL-13, IL-25, IL-33 and β-actin oligonucleotide coupled primers were synthesized by Integrated DNA Technologies (Coralvile, LA, USA). Lysis buffer for protein extraction was purchased from Jubiotech (Dajeon, Republic of Korea). Primary antibodies against rabbit p-STAT1, STAT1, p-STAT6, STAT6, p-STAT3, STAT3, and β-actin were purchased from Cell Signaling Technology^®^ (Danvers, MA, USA). All materials used in this study were of the highest available quality. 

### 2.3. High-Performance Liquid Chromatography (HPLC) Analysis

HPLC analysis of the LK5 herbal complex was performed using a Waters Alliance e2695 separation module coupled to a Waters 2998 photodiode array (PDA) detector. The HPLC column was a YMC J’sphere ODS-H80 column (4.6 × 250 mm, 4 μm) with an injection volume of 10 μL. Then, the temperature of the column oven was maintained at 35 °C, and the flow rate was set at 0.95 mL/min. The mobile phases used for the analysis were (A) water and (B) acetonitrile, with the following elution system: 0–5 min, (A) 92% (B) 8%; 5–10 min, (A) 86% (B) 14%; 10–19 min, (A) 74% (B) 26%; 19–30 min, (A) 71% (B) 29%; 30–35 min, (A) 32% (B) 68%; 35–44 min, (A) 0% (B) 100%; 44–46 min, (A) 92% (B) 8%; and 46–56 min, (A) 92% (B) 8%. The wavelength of the detector was set at 210 nm. The five standard compounds of oxymatrine, chlorogenic acid, baicalin, palmatine, and obacunone were obtained from the Natural Product Institute of Science and Technology (www.nist.re.kr, accessed on 11 December 2023, Anseong, Republic of Korea). Standard solutions were prepared by dissolving 1.5 mg of each of the five standard compounds in 80% methanol. Additionally, the LK5 herbal complex was dissolved in 80% methanol to a concentration of 50 mg/mL. Both the standard and the LK5 herbal complex solutions were filtered through a 0.45 μm PVDF membrane. The concentrations of the five standard compounds were determined by constructing a calibration curve using concentration (X, mg/10 μL) and peak area (Y), and the mean ± standard deviation was calculated.

### 2.4. Cell Culture

The HaCaT cells were donated by the food chemistry laboratory (Prof. O.H Lee) at Kangwon National University. The cell culture medium was used in DMEM with 100 units/mL P/S and 10% FBS. The cells were cultured at 37 °C and 5% CO_2_, with subculturing every three days.

### 2.5. Cell Viability and Nitrite Determination

The cell viability was estimated using the 3-(4,5-dimethylthiazol-2-yl)-2,5-diphenyltetrazolium bromide (MTT) assay. The HaCaT cells were treated with the LK5 herbal complex (1, 3, 10, and 30 mg/mL). Following a 4 h incubation with MTT solution (5 mg/mL in PBS) at 37 °C, the cells were subsequently treated with DMSO (100 μL/well) to solubilize the formazan crystals. The optical density was measured at 540 nm using a microplate spectrophotometer (Molecular Devices, Sunnyvale, CA, USA). The concentration of NO in the culture medium was determined as nitrite by the griess reagent. The concentration of nitrite was converted into the sodium nitrite concentration as a standard [25].

### 2.6. Reverse Transcription Quantitative Polymerase Chain Reaction (RT-qPCR)

The total RNA was extracted from the HaCaT cells and ear tissue using RNAiso Plus. The extracted total RNA was used to generate cDNA through reverse transcription with All-in-One first-strand cDNA synthesis SuperMix [26]. The QuantStudio 3 (Applied Biosystems, Foster City, CA, USA) system with PowerSYBR Green PCR Master Mix and gene-specific primers was utilized for RT–qPCR analysis, with the synthesized cDNA used as the template. The Ct value was determined and normalized to the average Ct value of the control gene (β-actin), and the relative expression was quantified with the 2^−ΔΔ^Ct method. Each primer sequence is shown in Table 1. The PCR analyses were conducted utilizing the following parameters: 40 cycles of 95 °C for 15 s, 57 °C for 20 s, and 72 °C for 40 s (IL-1β, IL-4, IL-6, IL-13, IL-25, IL-33, TNF-α, and iNOS) in the in vitro study; 40 cycles of 95 °C for 15 s, 50 °C for 10 min, and 70 °C for 10 min (TSLP, IL-4, and IL-13) in the in vivo study.

### 2.7. Enzyme-Linked Immunosorbent Assay (ELISA)

In the in vitro studies, the expressions of TNF-α, IL-1β, and IL-6 in the culture supernatants obtained from the HaCaT cells were evaluated using ELISA kits (R&D Systems, Minneapolis, MN, USA). The cells were pretreated with the LK5 herbal complex for 1 h at various concentrations (0.1, 0.3, 1, and 3 mg/mL) and subsequently stimulated with LPS (1 μg/mL) for 1 day. 

In the in vivo animal study, the blood was centrifuged (4 °C, 10,000 rpm, 5 min) in a heparinized tube. Supernatant from the blood was collected to obtain plasma. The TSLP, IgE, and IL-6 levels in the plasma were measured using an ELISA kit (R&D Systems, Minneapolis, MN, USA) according to the manufacturer’s protocol. 

### 2.8. Western Blot Analysis

The protein was isolated from the cells using lysis buffer (Jubiotech, Daejeon, Republic of Korea) with a protease phosphatase inhibitor cocktail (Thermo Fisher Scientific, Rockford, IL, USA). The proteins were quantified using a Bradford assay. The protein was subjected to 8% SDS-PAGE and transferred to a PVDF membrane. The membrane was incubated with blocking buffer for 2 h. Primary antibodies (p-STAT1, p-STAT3 = 1:500, p-STAT6 = 1:1000, STAT3, STAT1, STAT6, β-actin = 1:1000) were incubated overnight at 4 °C. Then, the membrane was washed five times with TBST. Finally, the membrane was incubated for 2 h with a secondary antibody at room temperature. Immunoblots were imaged using an ImageQuantTM LAS 500 (GE Healthcare, Chicago, IL, USA). Additionally, the immunoblots were analyzed using the Image J program (LOCI, University of Wisconsin, Madison, WI, USA) and the phosphorylation level was determined by calculating the ratio of phosphorylated protein to total protein on the same membrane.

### 2.9. DNCB-Induced Animal Model of Atopic Dermatitis

**Figure 1:**
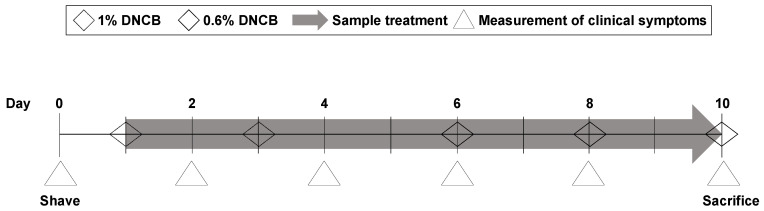
Time schedule for the DNCB-induced AD model. The mice were sensitized with 1% DNCB twice. Then, the mice were treated with the LK5 herbal complex (12.5, 25, 50 mg/kg and dexamethasone 1 mg/kg) for 10 days.

For the sensitization step, 200 μL of 1% 2,4-dinitrochlorobenzene (DNCB) solution dissolved in an acetone and olive oil mixture (3:1) was applied on the dorsal skin and 20 μL to the right ear. Various concentrations of the LK5 herbal complex (12.5, 25, 50 mg/kg, and dexamethasone 1 mg/kg) were administered daily after sensitization for 10 days, and a 0.6% DNCB solution was applied to the dorsal skin and right ear every other day to maintain symptoms (Figure 1). We assessed clinical symptoms every other day until the end of the experiment.

### 2.10. Measurement of Clinical Symptoms Including Dermatitis Score and Ear Thickness

The dermatitis score was evaluated based on the SCORAD index. Skin dermatitis was evaluated on the basis of 0–3 points according to the severity of the four items such as erythema/hemorrhage edema, erosion/excoriation, and dryness/lichenification (none—0, mild—1, moderate—2 and severe—3) [27]. The thickness of the right ear was measured using a digital micrometer (Mitutoyo, Kawasaki, Japan). The clinical symptom measurements were blinded, and the information regarding each experimental group was not disclosed.

### 2.11. Transepidermal Water Loss

To measure transepidermal water loss (TEWL) in the dorsal skin of AD mice, we used GPSKIN Barrier Pro (GPpower, Hanam, Republic of Korea) through the GPSKIN Research program. The TEWL was assessed every other day along with clinical symptoms.

### 2.12. Histological Analysis

The skin on the back of the mouse was collected with a biopsy punch and fixed in 10% formalin. The fixed tissue was cut into 5 μm thick sections and stained with hematoxylin and eosin (H&E) and toluidine blue (TB). For the histological analysis, images were obtained using a light microscope (Olympus, Tokyo, Japan). For epidermal thickness, the stained area was observed at a magnification of 200× and analyzed. The infiltration of mast cells was evaluated by counting the number of mast cells in two randomly selected sections.

### 2.13. Pruritus

Six-week-old male mice were purchased from KOATECH Inc. (Pyeongtaek, Republic of Korea). The LK5 herbal complex (12.5, 25, and 50 mg/kg) was orally administered for 7 days. The positive control terfenadine (histamine H1 receptor antagonist, 10 mg/kg) was orally administered 1 h before the behavior test. Pruritus was assessed for 30 min immediately after subcutaneous injection of compound 48/80 (50 µg/50 µL) on the final day of treatment with the LK5 herbal complex. The scratching and subsequent paw withdrawal moment of the itchy area on the back of the neck were evaluated as one event [28]. Additionally, the evaluation was conducted in a blinded manner, similar to the clinical symptom measurements.

### 2.14. Statistic

The statistical analyses were conducted with GraphPad Prism version 8.0 (GraphPad, La Jolla, CA, USA). The mean ± S.E.M. was used to present the experimental values. Two-way analysis of variance (ANOVA) was conducted for statistical analysis of the ear thickness, TEWL, and dermatitis score. One-way ANOVA was performed for RT-qPCR, ELISA, NO assay, MTT assay and pruritus, and histological analysis. When the data were significant, the Newman–Keuls test was used for multiple comparisons. Significance was defined as *p* < 0.05.

## 3. Results 

### 3.1. Quantification of the LK5 Herbal Complex Using HPLC

**Figure 2:**
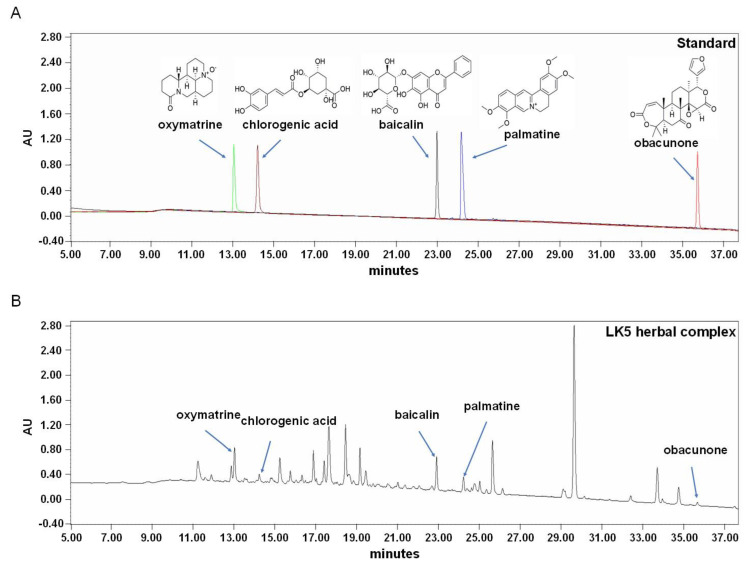
HPLC chromatograms of (**A**) the standard compounds and (**B**) the LK5 herbal complex. The indicator substances are oxymatrine, chlorogenic acid, baicalin, palmatine, and obacunone, which were analyzed sequentially via HPLC. Oxymatrine was separated sequentially on a YMC J’sphere ODS-H80 column (4.6 × 250 mm, 4 μm) at 13 min, with chlorogenic acid at 15 min 20 s, baicalin at 23 min, palmatine at 24 min 20 s, and obacunone at 36 min. The detection of the five standard compounds was confirmed by comparison with the standards of each compound.

The LK5 herbal complex contains *Scutellaria baicalensis*, *Liriope platyphylla*, *Sophora flavescens*, *Dictammus dasycarpus*, and *Phellodendron Schneider*. In the LK5 herbal complex, the standard compound for *S*. *baicalensis* is baicalin, while for *S*. *flavescens*, the standard compound is oxymatrine [29,30]. Additionally, the standard compound for *P. Schneider* is palmatine, and for *D. dasycarpus* and *L. platyphylla,* the standard compounds are obacunone and chlorogenic acid, respectively [22,31,32]. The five standard compounds were detected through HPLC chromatograms (Figure 2, Table 2). Based on the HPLC results, it was determined that the LK5 herbal complex has anti-inflammatory properties due to its high content of baicalin and oxymatrine [33,34]. Specifically, we can speculate that the five standard compounds known for their anti-inflammatory properties may exhibit even more potent anti-inflammatory effects when combined.

### 3.2. The LK5 Herbal Complex Inhibited LPS-Stimulated Inflammatory Mediators and Inflammatory Cytokines in HaCaT Cells

**Figure 3:**
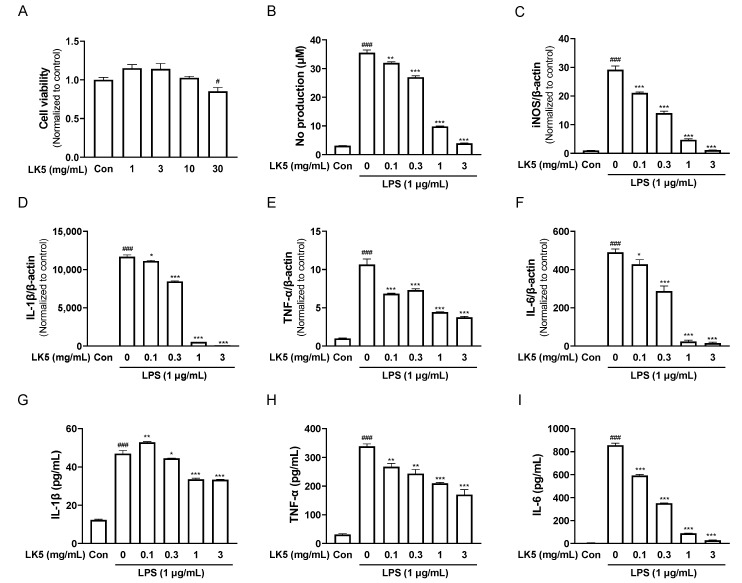
Anti-inflammatory effects of the LK5 herbal complex in LPS-stimulated HaCaT cells. (**A**) Cell viability. (**B**) NO production. (**C**–**F**) iNOS, IL-1β, TNF-α, and IL-6 mRNA expressions via RT-qPCR. (**G**–**I**) IL-1β, TNF-α, and IL-6 production via ELISA. The data were determined after LPS stimulation for 24 h. Statistical analysis was performed using one-way ANOVA. The data represent the means ± S.E.M. ^#^ *p* < 0.05, ^###^ *p* < 0.001 vs. the Con group, * *p* < 0.05, ** *p* < 0.01, *** *p* < 0.001 vs. the 0 (LPS-stimulated) group. Con, control; LK5, LK5 herbal complex.

HaCaT cells are human-derived keratinocytes with properties similar to human keratinocytes [35]. It is also known that HaCaT cells can secrete inflammatory cytokines by stimuli and express inflammation-related genes to induce or modulate inflammatory responses such as AD [36]. Therefore, we determined the anti-inflammatory effects of the LK5 herbal complex in HaCaT cells stimulated with LPS. First, we conducted an MTT assay to determine the cytotoxic concentrations of the LK5 herbal complex. At both 1–30 mg/mL concentrations, cell viability was not reduced by the LK5 herbal complex treatment (Figure 3A). Next, HaCaT cells were stimulated with 1 μg/mL LPS for 24 h after treatment with the LK5 herbal complex at each concentration for 1 h. The LK5 herbal complex reduced NO production in a concentration-dependent manner and inhibited the expression of iNOS mRNA, which is involved in NO production and synthesis (Figure 3B,C). LPS is known to stimulate the production of inflammatory cytokines such as TNF-α, IL-1β, and IL-6 [37,38,39]. Therefore, we investigated whether the LK5 herbal complex inhibits inflammatory cytokine production. Treatment with the LK5 herbal complex significantly reduced the LPS-induced production of inflammatory cytokines such as IL-1β, TNF-α, and IL-6 (Figure 3D–F). Additionally, the LK5 herbal complex inhibited the production of IL-1β, TNF-α, and IL-6 at the protein level (Figure 3G–I). All of the results suggest that the LK5 herbal complex exerts anti-inflammatory effects by inhibiting NO production and inflammatory cytokine expression in HaCaT cells.

### 3.3. The LK5 Herbal Complex Downregulated the Phosphorylation of STAT in LPS-Stimulated HaCaT Cells

**Figure 4:**
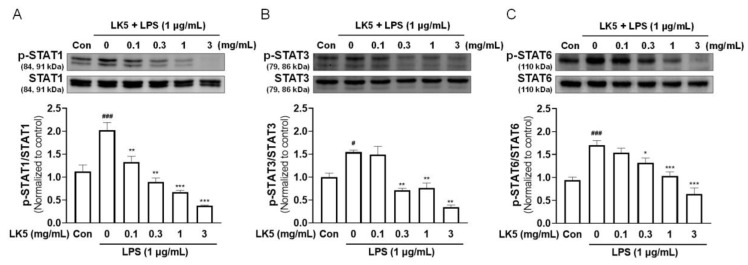
Effects of the LK5 herbal complex on LPS-stimulated STAT signaling in HaCaT cells. HaCaT cells were pretreated with the LK5 herbal complex for 1 h, and then exposed to 1 μg/mL LPS for 3 h. The treated cells were assessed for activation of (**A**) STAT1, (**B**) STAT3, and (**C**) STAT6 expressions using Western blot analysis. Statistical analysis was performed using one-way ANOVA. The data represent the means ± S.E.M. ^#^ *p* < 0.05, ^###^ *p* < 0.001 vs. the Con group, * *p* < 0.05, ** *p* < 0.01, *** *p* < 0.001 vs. the 0 (LPS-stimulated) group. Con, control; LK5, LK5 herbal complex.

Increasing inflammatory cytokines by LPS can activate the JAK-STAT pathway [40,41]. In particular, when STAT1, STAT3, and STAT6 are activated as transcription factors by inflammatory cytokines, they can induce the expressions of genes and lead to inflammatory conditions such as asthma, rheumatoid arthritis, and AD [42,43]. As shown in Figure 3D–I, we found that the LK5 herbal complex effectively reduced the inflammatory cytokines increased by LPS in HaCaT cells. Therefore, we evaluated the effect of the LK5 herbal complex on the changes in transcription factor STAT phosphorylation in LPS-stimulated HaCaT cells. The LPS-induced increase in STAT1, STAT3, and STAT6 phosphorylation was dose-dependently reduced by LK5 herbal complex treatment in LPS-stimulated HaCaT cells (Figure 4A–C). These results suggest that the LK5 herbal complex may regulate inflammation by modulating the transcription factor STAT.

### 3.4. The LK5 Herbal Complex Regulated Inflammatory Cytokines by Inhibiting STAT Phosphorylation in IL-4/IL-13-Stimulated HaCaT Cells

**Figure 5:**
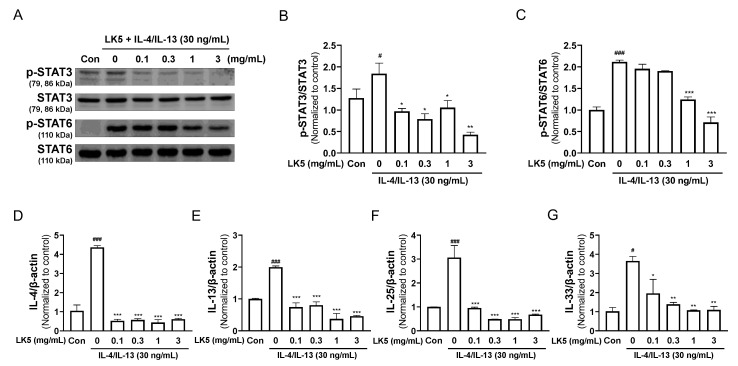
Effects of the LK5 herbal complex on IL-4/IL-13-stimulated STAT signaling and inflammatory cytokines in HaCaT cells. HaCaT cells were pretreated with the LK5 herbal complex for 1 h, and then exposed to IL-4/IL-13 (30 ng/mL) for 45 min. The treated cells were assessed for (**A**,**B**) STAT3, and (**A**,**C**) STAT6 expression using Western blot analysis. HaCaT cells were pretreated with the LK5 herbal complex for 1 h, and then exposed to IL-4/IL-13 (30 ng/mL) for 24 h. The LK5 herbal complex-treated cells were evaluated for AD-related inflammatory cytokines such as (**D**) IL-4, (**E**) IL-13, (**F**) IL-25, and (**G**) IL-33 mRNA expressions via RT-qPCR. Statistical analysis was performed using one-way ANOVA. The data represent the means ± S.E.M. ^#^ *p* < 0.05, ^###^ *p* < 0.001 vs. the Con group, * *p* < 0.05, ** *p* < 0.01, *** *p* < 0.001 vs. the 0 (IL-4/IL-13-stimulated) group. Con, control; LK5, LK5 herbal complex.

Activation of the transcription factor STAT is involved in various inflammatory diseases and, in particular, STAT3 and STAT6 are known to play a role in exacerbating AD [44]. The IL-4, IL-13, IL-25, and IL-33 that are expressed in response to the transcription of STAT3 and STAT6, have been implicated in the exacerbation of AD, and are known to be increased in AD patients [45,46,47,48]. Therefore, given that the LK5 herbal complex decreased the phosphorylation of STAT in LPS-stimulated HaCaT cells, we further investigated whether the LK5 herbal complex shows similar effects after stimulation with IL-4/IL-13. The LK5 herbal complex downregulated IL-4/IL-13-induced phosphorylation of STAT3 and STAT6 (Figure 5A–C). Additionally, the LK5 herbal complex dose-dependently decreased the expressions of the inflammatory cytokines such as IL-4, IL-13, IL-25, and IL-33, which are regulated by STAT3 and STAT6 (Figure 5D–G). These results demonstrate that the LK5 herbal complex reduces the expression of inflammatory cytokines in IL-4/IL-13-stimulated HaCaT cells by inhibiting STAT signaling.

### 3.5. The LK5 Herbal Complex Ameliorated Clinical Symptoms and Histological Analysis in DNCB-Induced AD and Compound 48/80-Induced Pruritis Animal Models

**Figure 6:**
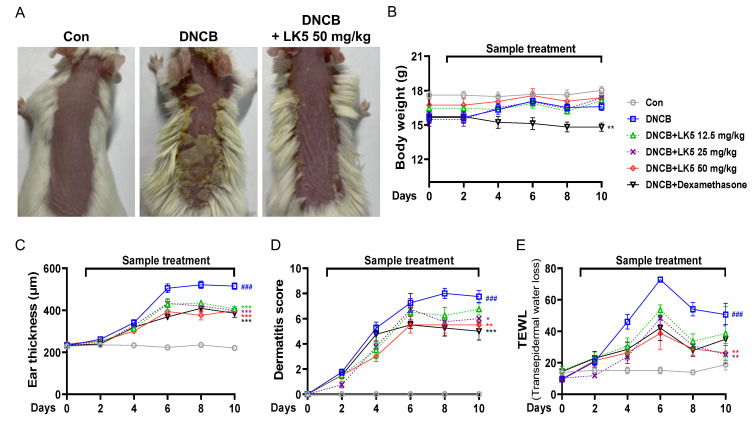
Effects of the LK5 herbal complex on clinical symptoms in DNCB-induced skin lesions. (**A**) Images of the dorsal skin were taken on day 10, and are shown as representative images for group. Every 2 days, the clinical symptoms, including (**B**) body weight, (**C**) ear thickness (µm), (**D**) dermatitis score, and (**E**) TEWL, were assessed (*n* = 8). Statistical analysis was performed using two-way ANOVA. The data represent the means ± S.E.M. ^###^ *p* < 0.001 vs. the control group, * *p* < 0.05, ** *p* < 0.01, *** *p* < 0.001 vs. the DNCB-only group. Con, control; Dexa, dexamethasone; LK5, LK5 herbal complex.

Based on the anti-inflammatory effects of the LK5 herbal complex in HaCaT cells, we further evaluated whether the LK5 herbal complex exhibits anti-AD effects in a DNCB-induced AD-like lesion model. After inducing AD-like lesions with DNCB, the LK5 herbal complex was administered orally for 10 days to assess improvement in critical AD symptoms such as dermatitis, epidermal water loss, and infiltration of mast cells [49]. While the LK5 herbal complex treatment showed no decrease in mouse body weight, dexamethasone reduced body weight compared to the DNCB-only group (Figure 6B). The dermatitis score, ear thickness, and TEWL of the DNCB-only group were increased compared to those of the control group (Figure 6A). However, we perceived that the ear thickness and dermatitis score were dose-dependently decreased by the LK5 herbal complex compared to the DNCB-only group (Figure 6C,D). In addition, administration of the LK5 herbal complex reduced the TEWL in comparison with the DNCB-only group (Figure 6E). 

**Figure 7:**
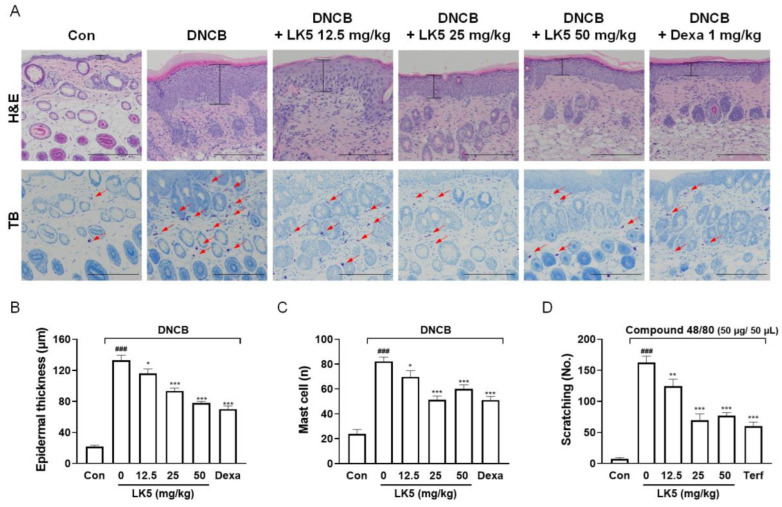
Effects of the LK5 herbal complex on histological changes in DNCB-induced skin lesions and itching in compound 48/80-induced mice. (**A**) Dorsal skin images stained with H&E and TB were captured at 200× magnification (scale bar: 200 μm). Analysis was conducted on (**B**) epidermal thickness (black line), and (**C**) mast cell infiltration (red arrows) was assessed (*n* = 4). (**D**) Changes in pruritus with the LK5 herbal complex administration following compound 48/80 treatment. Statistical analysis was performed using two-way ANOVA. The data represent the means ± S.E.M. ^###^ *p* < 0.001 vs. the control group, * *p* < 0.05, ** *p* < 0.01, *** *p* < 0.001 vs. the DNCB- and compound 48/80-only group. Con, control; Dexa, dexamethasone; Terf, terfenadine, LK5; LK5 herbal complex.

AD is characterized by increased skin thickness in the lesioned area and increased infiltration of mast cells that express itch-inducing substances [9,15]. We found that the LK5 herbal complex reduced the epidermal thickness and the number of infiltrated mast cells in mice in a concentration-dependent manner (Figure 7A–C). Since the LK5 herbal complex reduced mast cells, we also investigated whether it also affected itching behavior, a common symptom of AD [50]. In the itching behavior test, the compound 48/80-only group exhibited vigorous scratching behavior compared to the control group. However, the LK5 herbal complex or the positive control terfenadine effectively inhibited scratching behavior induced by compound 48/80 at all concentrations (Figure 7D). These results suggest that the LK5 herbal complex improves the clinical symptoms and histologic changes of AD and inhibits pruritus, which a typical symptom of AD. 

### 3.6. The LK5 Herbal Complex Inhibited IgE and Inflammatory Cytokines in the DNCB-Induced AD Animal Model

**Figure 8:**
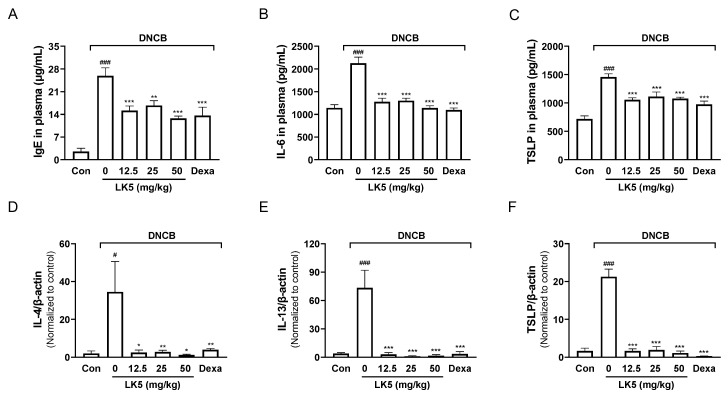
Effect of the LK5 herbal complex on the levels of IgE and inflammatory cytokines, including IL-4, IL-13, and TSLP, in AD-like lesioned mice. The plasma protein levels of (**A**) IgE, **(B**) IL-6, and (**C**) TSLP were determined using an ELISA kit (*n* = 6). The mRNA expressions of (**D**) IL-4 (**E**) IL-13, and (**F**) TSLP in DNCB-induced ear tissue were evaluated via RT-qPCR. Statistical analysis was performed using one-way ANOVA. The data represent the means ± S.E.M. ^#^ *p* < 0.05, ^###^ *p* < 0.001 vs. the control group, * *p* < 0.05, ** *p* < 0.01, *** *p* < 0.001 vs. the DNCB-only group. Con, control; Dexa, dexamethasone.

IgE, IL-6, and TSLP, which are known to be elevated in AD patients, play a central role in exacerbating AD symptoms [6,7]. In the plasma of the DNCB-induced AD-like animal models, the LK5 herbal complex treatments significantly inhibited TSLP, IL-6, and IgE levels compared to the DNCB-only group (Figure 8A–C). These data indicate that downregulating the inflammatory cytokines TSLP, IL-6, and IgE could be an important strategy in the treatment of AD. 

Inflammatory cytokines such as TSLP, IL-4, and IL-13 can activate inflammatory cells in skin tissues, causing edema and worsening symptoms, especially in AD patients [51,52,53]. To determine whether AD symptoms were improved by inhibition of inflammatory cytokines, we evaluated whether the LK5 herbal complex reduces inflammatory cytokines in DNCB-induced lesional ear tissue. We found that DNCB-induced increases in inflammatory cytokines, including IL-4, IL-13, and TSLP, were fully inhibited by the LK5 herbal complex in lesional ear tissue (Figure 8D–F). These results demonstrate that the LK5 herbal complex effectively suppresses the increased inflammatory cytokines in DNCB-induced ear tissue, ultimately alleviating AD-like symptoms such as edema.

## 4. Discussion

AD is a chronic inflammatory skin disease that typically occurs in early childhood, but it has recently been recognized as having a high prevalence in adults [2]. AD is characterized by a vicious itching-scratching cycle, and is believed to be caused by a combination of genetic and environmental factors [4,5]. AD is caused by the release of TSLP and IL-4/IL-13 from T cells by foreign antigens, activating the JAK-STAT pathway [44]. The subsequent transcription of STAT produces inflammatory cytokines, which can induce inflammation and itching [11,12]. This process disrupts the skin barrier and ultimately contributes to the onset and worsening of AD [54]. Current treatments for AD aim to improve the outward phenotype rather than cure it [55]. Steroids and antihistamines are currently the most common treatments for AD due to their role in reducing skin inflammation and relieving itching, but they can be associated with side effects such as growth retardation and osteoporosis [17,18]. 

In this study, we used the LK5 herbal complex, a combination of five plants including *Scutellaria baicalensis*, *Liriope platyphylla*, *Sophora flavescens*, *Dictammus dasycarpus*, and *Phellodendron Schneider*. *S*. *baicalensis* exhibited anti-AD effects in an IgE-induced murine allergy model by suppressing histamine expression, Mitogen-Activated Protein Kinase (MAPK) phosphorylation, and inflammatory cytokine expression [56,57]. *L*. *platyphylla* alleviated ear swelling and inhibited IgE expression and inflammatory cytokines in a phthalic anhydride-induced mouse model of AD [58,59]. Similarly, *S*. *flavescens* relieved scratching behavior in serotonin-induced itching in mice [60,61]. *D*. *dasycarpus* has demonstrated anti-psoriatic effects in an imiquimod-induced murine model, reducing inflammatory cytokine expression by inhibiting STAT3 phosphorylation [61,62,63]. *P*. *Schneider* has demonstrated anti-inflammatory effects in an LPS-induced sepsis model, inhibiting the phosphorylation of MAPK, thereby suppressing the expression of inflammatory cytokines in the liver [64]. Therefore, we assume that the five plants in the LK5 herbal complex possess anti-inflammatory properties. In addition, baicalin, a standard compound of *S*. *baicalensis*, has been shown to exhibit anti-AD in a DNCB-induced AD model by inhibiting JAK-STAT phosphorylation, reducing inflammatory cytokines [29,65,66]. Chlorogenic acid, a standard compound of *L*. *platyphylla*, alleviated pruritus in surfactant-induced mice by inhibiting histamine secretion, and reduced increasing eosinophils and lymphocytes induced by ovalbumin [67,68,69]. Furthermore, oxymatrine, a representative standard compound of *S*. *flavescens*, inhibited JAK-STAT phosphorylation and decreased the expression of inflammatory cytokines in MC903-induced AD mice [30,70]. Obacunone, a standard compound of *D*. *dasycarpus*, demonstrated anti-inflammatory efficacy by reducing inflammatory cytokines such as IL-6 in CCl4-induced liver fibrotic mice [31,71]. Palmatine, the representative compound of *P*. *Schneider*, alleviated skin lesions in ovalbumin-induced mice of urticaria, and alleviated itching by inhibiting the infiltration of mast cells [72,73]. Thus, these compounds promote immunological control and inhibit the inflammatory response, eventually relieving symptoms, including skin rashes and itching [31,34,65,66,68,71,72]. In the present study, we found that the LK5 herbal complex effectively ameliorated DNCB-induced AD-like lesions without side effects such as weight loss. In addition, the LK5 herbal complex inhibited inflammatory cytokines, which can aggravate inflammation and attenuate itching. These results were attributed to the combined anti-inflammatory effects of oxymatrine, chlorogenic acid, baicalin, palmatine, and obacunone, which are the indicator substances of the LK5 herbal complex. Therefore, the LK5 herbal complex may provide new research material for the prevention, treatment, and alleviation of AD with fewer side effects, such as weight loss, and is safer.

This study observed the anti-inflammatory properties of the LK5 herbal complex in HaCaT cells stimulated with LPS and IL-4/IL-13. Inflammatory cytokines such as TNF-α, IL-6, and IL-1β are increased by LPS, which is known to promote inflammatory responses such as vasodilation and immune cell migration [37,38,39]. Recently, studies have shown that these cytokines can regulate the transcription factor STAT, which expresses several inflammatory genes [74,75]. In LPS-induced HaCaT cells, we found that the LK5 herbal complex inhibited TNF-α, IL-1β, and IL-6 expression levels and reduced phosphorylation of STAT1, STAT3, and STAT6. In addition, STAT3 and STAT6 are also activated by IL-4/IL-13, which has been implicated in the AD immune response [10]. In this study, the LK5 herbal complex inhibited the phosphorylation of STAT3 and STAT6 that was increased by IL-4/IL-13, which also reduced the subsequent expression of inflammatory cytokines such as IL-4, IL-13, IL-25, and IL-33. Mounting evidence has reported that regulating STAT and inflammatory cytokines can modulate the activity of immune cells and suppress immune response imbalance, thereby reducing inflammation [76,77]. These previous studies may support our results that the LK5 herbal complex has anti-inflammatory properties. 

We also found that the LK5 herbal complex suppressed IgE, TSLP, and IL-6 in plasma, and increased epidermal thickness and mast cell infiltration in a DNCB-induced AD-like lesion model. In the case of the epidermis, the thickness increases are known to result from inflammatory factors and migration of immune cells [15]. Therefore, inhibiting the expressions of inflammatory cytokines such as TSLP and IL-6 may have anti-inflammatory properties that reduce epidermal thickness, alleviating edema. In addition, histamine released by mast cells is known to cause itching, a typical symptom of AD [6]. Therefore, the decrease in scratching behavior can be attributed to the suppression of histamine. Previous studies have shown that injection of compound 48/80 into mice increases the expression of histamine, resulting in increased scratching behavior [78]. The LK5 herbal complex was shown to attenuate itching behavior induced by the histamine derivative compound 48/80. Thus, the LK5 herbal complex appears to have antipruritic activity by inhibiting histamine release in mast cells.

Notably, IL-4 and IL-13 are known to upregulate other inflammatory cytokines, promoting inflammatory responses such as edema and contributing to AD exacerbation [79,80]. Additionally, anti-IL-4/IL-13 monoclonal antibodies have been used for the treatment of AD patients in clinics [10]. Therefore, modulating IL-4 and IL-13 may improve AD by suppressing the inflammatory response. This study showed that the LK5 herbal complex inhibited IL-4 and IL-13 in DNCB-induced mouse ear tissue. This evidence supports the notion that the LK5 herbal complex has anti-AD effects by alleviating edema through its role in regulating the expression of inflammatory cytokines as well as reducing vascular permeability at the lesional site. 

In conclusion, the LK5 herbal complex has anti-inflammatory properties that reduce LPS- and IL-4/IL-13-induced inflammation, and has anti-AD effects in animal models of DNCB-induced AD and compound 48/80-induced pruritus. Accordingly, we suggest that the LK5 herbal complex has high potential for development as a natural AD treatment. These findings provide a new direction for treating AD, and are recognized as an important study that opens up the possibility of clinical applications of the LK5 herbal complex. Further mechanistic studies are needed to determine whether the LK5 herbal complex also inhibits STAT and other signaling pathways upstream of inflammatory cytokines in DNCB-induced animal models.

## Figures and Tables

**Table 1:** Primer sequences.

Target Gene	Primer Sequence
*IL-1β*	F 5′-ACCT GCT GGT GTG TGA CGT T-3′ R 5′-TCG TTG CTT GGT TCT CCT TG-3′
*IL-6*	F 5′-GAG GAT ACC ACT CCC AAC AGA CC-3′ R 5′-AAG TGC ATC ATC GTT GTT CAT ACA-3′
*TNF-α*	F 5′-AAATGGGCTCCCTCTCATCAGTTC-3′ R 5′-TCTGCTTGGTGGTTTGCTACGAC-3′
*iNOS*	F 5′-CAT GCT ACT GGA GGT GGG TG-3′ R 5′-CAT TGA TCT CCG TGA CAG CC-3′
*TSLP*	F 5′-GGA CCA CTG GTG TTT ATT CT-3′ R 5′-CGA GGT TTA GAT GCT GTC AT-3′
*IL-4*	F 5′-AGA TGG ATG TGC CAA ACG TCC TCA-3′ R 5′-AAT ATG CGA AGC TTG GAA GCC-3′
*IL-13*	F 5′-GCA ACG GCA GCA TGG TAT GGA-3′ R 5′-TGG TAT AGG GGA GGC TGG AGA C-3′
*IL-25*	F 5′-ACA GGG ACT TGA ATC GGG TC-3′ R 5′-TGG TAA AGT GGG ACG GAG TTG-3′
*IL-33*	F 5′-CAC ATT GAG CAT CCA AGG AA-3′ R 5′-AAC AGA TTG GTC ATT GTA TGT ACT CAG-3′
*β-actin*	F 5′-ATC ACT ATT GGC AAC GAG CG-3′ R 5′-TCA GCA ATG CCT GGG TAC AT-3′

**Table 2:** The contents of the LK5 herbal complex of indicator substances measured with HPLC.

Standard Compounds	Oxymatrine	Chlorogenic Acid	Baicalin	Palmatine	Obacunone
**Content** **(mg/g)**	2.52 ± 0.00	0.40 ± 0.00	2.61 ± 0.01	0.75 ± 0.00	0.36 ± 0.00

## Data Availability

Data will be made available on request.

## Abbreviations

AD	Atopic dermatitis
DNCB	2,4-Dinitrochlorobenzene
ELISA	Enzyme-linked immunosorbent assay
H&E	Hematoxylin and eosin
IL	Interleukin
IgE iNOS	Immunoglobulin E Inducible nitric oxide synthase
LPS MAPK NO	Lipopolysaccharide Mitogen-Activated Protein Kinase Nitric oxide
RT-qPCR	Reverse transcription-quantitative polymerase chain reaction
STAT	Signal transducers and activators of transcription
TB TEWL TNF-α TSLP	Toluidine blue Transepidermal water loss Tumor necrosis factor-α Thymic stromal lymphopoietin
